# Disparities in Access and Utilization of Services for Older Adults in Connecticut’s MFP program

**DOI:** 10.1093/geroni/igaf122.331

**Published:** 2025-12-31

**Authors:** Asmita Aasaavari, Ellis Dillon, Kathy Kellett, Christine Bailey, Amal Alsamawi, Richard Fortinsky, Lisa Barry, Julie Robison

**Affiliations:** University of Connecticut, Farmington, Connecticut, United States; University of Connecticut, Farmington, Connecticut, United States; University of Connecticut, Farmington, Connecticut, United States; University of Connecticut, Farmington, Connecticut, United States; University of Connecticut, Farmington, Connecticut, United States; University of Connecticut, Farmington, Connecticut, United States; University of Connecticut, Farmington, Connecticut, United States; University of Connecticut, Farmington, Connecticut, United States

## Abstract

Money Follows the Person (MFP) is a Medicaid program that helps older adults move from Long-Term Care institutions to the community. In this study, we examine the mechanisms that contribute to disparities for older persons living with dementia (PLWD) in access to, utilization of, and quality of the MFP program in Connecticut. This qualitative study thematically analyzes in-depth interviews and focus groups with MFP-involved professionals (n = 23), family caregivers (n = 14), and older adults (n = 24) who (1) never applied to MFP, (2) applied but never moved, (3) applied, moved, and sustained community-living, and (4) moved but were reinstitutionalized. Dominant themes include: (1) Mismatch between many PLWDs’ care needs and MFP program resources: participants described how MFP budgets, services, and training were not designed to address the multiple supports many PLWDs need. (2) Importance of self/family-advocacy: Learning about the MFP program, completing the application, and arranging a move required advocacy to make calls, assemble paperwork, find housing, and have family members provide informal support. Despite Connecticut’s longstanding MFP program, interviews and focus groups underscored how program procedures pose additional obstacles for PLWD and their families. Recommendations for innovative solutions include upstream planning upon nursing home admission, re-designing siloed and inflexible HCBS programs, leveraging new technologies (e.g., remote monitoring/supports), expanding assistive technology, and developing supportive housing to better integrate PLWD into communities. Identifying better solutions for community living and improving concordance between the design of MFP and HCBS programs are paramount in meeting the unique needs of PLWD who are transitioning to the community.

